# A structured approach to neurologic prognostication in clinical cardiac arrest trials

**DOI:** 10.1186/1757-7241-21-45

**Published:** 2013-06-10

**Authors:** Tobias Cronberg, Janneke Horn, Michael A Kuiper, Hans Friberg, Niklas Nielsen

**Affiliations:** 1 Department of Clinical Sciences, Lund University, Lund, Sweden; 2 Department of Neurology, Skane University Hospital, Lund, Sweden; 3 Department of Intensive Care, Academic Medical Center, Amsterdam, The Netherlands; 4 Department of Intensive Care Medicine, Medical Center Leeuwarden, Leeuwarden, The Netherlands; 5 Department of Intensive and Perioperative Care, Skane University Hospital, Lund, Sweden; 6 Department of Anesthesia and Intensive Care, Helsingborg University Hospital, Helsingborg, Sweden

**Keywords:** Cardiac arrest, Prognosis, Hypothermia, Target temperature

## Abstract

Brain injury is the dominant cause of death for cardiac arrest patients who are admitted to an intensive care unit, and the majority of patients die after withdrawal of life sustaining therapy (WLST) based on a presumed poor neurologic outcome. Mild induced hypothermia was found to decrease the reliability of several methods for neurological prognostication. Algorithms for prediction of outcome, that were developed before the introduction of mild hypothermia after cardiac arrest, may have affected the results of studies with hypothermia-treated patients. In previous trials on neuroprotection after cardiac arrest, including the pivotal hypothermia trials, the methods for prognostication and the reasons for WLST were not reported and may have had an effect on outcome. In the Target Temperature Management trial, in which 950 cardiac arrest patients have been randomized to treatment at 33°C or 36°C, neuroprognostication and WLST-decisions are strictly protocolized and registered. Prognostication is delayed to at least 72 hours after the end of the intervention period, thus a minimum of 4.5 days after the cardiac arrest, and is based on multiple parameters to account for the possible effects of hypothermia.

## Introduction

Out-of-hospital cardiac arrest (OHCA) is common and has an annual incidence of approximately 40–50 per 100.000 inhabitants in Europe and the US [1,2]. Despite an increased survival from all-rhythm OHCA [3,4] the overall mortality is still approximately 90% and at least 50% for those admitted to hospital but varies considerably between centers [5,6].

Following one randomized [7] and one quasi-randomized trial [8], mild systemic hypothermia was included in international guidelines as a recommended therapy for patients in coma after cardiac arrest [9,10]. However, a systematic review of previous trials concluded that the evidence in favor of hypothermia is weak and that earlier trials were associated with substantial risk of potential systematic and random error, as well as investigating only a selected patient population [11].

Brain injury is the dominant cause of death for patients who are admitted to the intensive care unit (ICU) following cardiac arrest [12,13] and a neurologic assessment of prognosis constitutes the foundation for decisions concerning limitations of care and interventions.

A clinical neurologic examination is usually combined with neurophysiologic, neuroradiologic and occasionally biochemical investigations to estimate the extent of brain injury. The predictive values of the different methods have been investigated in numerous trials and incorporated into clinical guidelines [9,14-16].

Mild hypothermia was found to alter the predictive value of several prognostic markers [17-20]. Earlier guidelines do not provide specific instructions on how to evaluate the hypothermia-treated patients [15] but more recent guidelines recommend delayed prognostication [4,16], based on multiple instruments [9,16].

From several recent studies on neurological prognostication after cardiac arrest it has become evident that the majority of deaths occur after a statement of a poor prognosis and withdrawal of life supportive treatment (WLST) [12,17-22]. Therefore, the routines for prognostication clearly have the power to affect survival rates in clinical trials. This is particularly relevant when blinding is difficult, as with different temperature regimes.

In this review we will discuss how mild hypothermia may affect neuroprognostication and in particular how the lack of protocols for neuroprognostication may have affected the results of previous cardiac arrest trials. Finally, we present how this issue is dealt with in a large ongoing trial: Target Temperature Management after Out-of-hospital Cardiac Arrest (the TTM-trial) (NCT01020916).

### Neuroprognostication after cardiac arrest

The majority of patients who are admitted to the ICU following cardiac arrest are unconscious [24]. Patients who improve their level of consciousness after withdrawal of sedative and analgesic substances usually have a good outcome [25]. For those who remain in coma, the prognosis becomes gradually worse with increasing time from the insult [26,27]. Repeated clinical neurologic examinations in combination with electrophysiologic investigations (electroencephalography (EEG) and somatosensory-evoked potentials (SSEP)) constitute the foundation of neurological prognostication. This may be further supported by neuroradiologic examinations (computed tomography and magnetic resonance imaging) and biomarkers (neuron specific enolase in particular), but the evidence for these methods is less solid [9,15,16]. Since the specificity of clinical as well as neurophysiologic findings increases with time, a well-founded judgment of prognosis can usually be made at 72 hours after cardiac arrest in a patient who has *not* been treated with hypothermia [15,26].

Hypothermia treatment makes the clinical neurological examination less reliable [17,20,22] and this may be, at least partly, related to an increased use [22] and decreased clearance [28,29] of sedative medication.

SSEP is less influenced by sedative medication than EEG and a bilateral loss of the cortical N20-potential at 24 hours or more after cardiac arrest, predicts a poor outcome with high accuracy [23,30]. The high specificity of SSEP appears to be retained for hypothermia-treated patients if the examination is performed after rewarming, but sporadic false predictions have been reported during hypothermia [19], and even after rewarming [31]. Furthermore, interobserver variability in the interpretation of SSEP has been reported [32].

Severely pathological EEG-patterns including burst-suppression, generalized status epilepticus and α-coma are associated with a poor prognosis after cardiac arrest. The EEG-pattern is sensitive to sedative medication and false predictions may occur [15]. For example, the occurrence of postanoxic status epilepticus has been reported in some patients with good outcome following cardiac arrest and induced hypothermia [33].

A status myoclonus, defined as generalized myoclonic seizures for more than 30 minutes and usually engaging facial and axial limb musculature, has been considered a reliable predictor of a poor prognosis if it occurs within 24 hours after cardiac arrest of a primary cardiac origin [34]. However, survival with good outcome despite early status myoclonus has been reported in hypothermia-treated patients [35].

A small fraction of cardiac arrest patients develops a total brain infarction with massive edema leading to herniation and complete loss of brain stem function [12,22]. Recently, a case report has questioned the accuracy of clinical tests to diagnose brain death in cardiac arrest survivors treated with hypothermia [36].

### Neuroprognostication and outcome in clinical trials after cardiac arrest

The Brain Resuscitation after Cardiac Arrest Trial (BRCT) 1 [37] and BRCT II [38] studies were conducted prior to the introduction of hypothermia and included comatose adult cardiac arrest patients. The BRCT I studied the effect of thiopentone and BRCT II the calcium entry-blocker lidoflazine. Neither study describes any rules for treatment decisions in patients who remained in coma. In 1994, Edgren et al. used the 262 control patients from the BRCT I study to investigate whether it was possible to reliably predict a permanent vegetative state few days after cardiac arrest. Variation in treatment of patients who remained in coma existed in this group, as the authors report: “For ethical and economic reasons it was not possible to require indefinite intensive care in the protocol, and local variations in decision making were permitted. Although most patients were given unlimited therapy, some in the Scandinavian centers were changed to intermediate care as early as 2–5 days after cardiac arrest.” No further details on treatment limitation or WLST were reported [26].

In 2002, the Hypothermia after Cardiac Arrest (HACA) [7] group and Bernard et al. [8] reported on the positive effects of treatment with hypothermia after cardiac arrest. After enrolment of 275 patients, the HACA study stopped inclusion due to a lower than expected inclusion rate. Of the included patients, 132 died during follow-up, but there is no information on how decisions on treatment limitations or withdrawal of care were made.

In the Bernard study, active life support was withdrawn from most patients who remained deeply comatose at 72 hours and this may actually have decreased the treatment effect since hypothermia makes the clinical examination less reliable at this point [17,20,22] and care may therefore have been withdrawn prematurely in cooled patients with a potential for late recovery [39]. On the other hand, the authors declare that “patients with an uncertain prognosis underwent tracheostomy” and this possibility to allow patients a prolonged observation period may have introduced a bias in favor of hypothermic treatment since the treating physicians were not blinded for temperature. The same holds true for decisions on WLST in both the Bernard and the HACA study. Of the 77 included patients in the Bernard study, 45 died during follow-up and the cause of death was severe neurologic injury and withdrawal of active therapy in the majority (34/45), which is similar to more detailed studies on the cause of death after CA [12,13]. The authors do not report which diagnostic methods were used in the study or how the results of investigations were weighed in treatment decisions. However, they state in the discussion that “because it was not feasible to blind clinicians to the patients’ treatment-group assignments, there is a possibility that bias affected patient care and outcome state” [8].

Another recent study, comparing hemofiltration with or without additional cooling versus control (no hemofiltration, no cooling), presents no information on how prognostication was performed or if decisions on treatment limitations and WLST were made [40].

Finally, a pilot study on the effect of high dosed erythropoietin was presented, but nothing is reported about treatment decisions and prognostication. Moreover, it is not evident from the protocol of the ensuing phase III study (http://www.controlled-trials.com/mrct/trial/706537) what diagnostics are used for prognostication and whether there are rules for WLST.

### The Target Temperature Management Trial (TTM)

In an attempt to explore the optimal target temperature management strategy for comatose survivors after cardiac arrest, the TTM-trial was launched in November 2010. In the trial, two strict target temperatures of 33°C and 36°C for 24 hours are compared. Primary outcome is survival until the end of trial [41]. The sample size of 950 adult patients with cardiac arrest of presumed cardiac origin was reached in January 2013, and the trial will be finished in mid 2013 after the stipulated 180-day evaluation. A standardized and transparent protocol for prognostication and WLST was regarded as a key component in the trial design, in order to reduce the risk of self-fulfilling prophecy and consequent bias.

A manual is available and investigators have been instructed concerning principles of prognostication at investigator meetings. The person who performs the prognostication is blinded to the intervention and all patients are regarded as if they were treated with hypothermia to 33°C. As a general principle, all patients in the trial are actively treated until a minimum of 72 hours after the end of the intervention period. Neurological prognostication is performed at this time-point or later for all patients who remain unconscious.

An earlier prognostication is allowed if (1) the patient becomes brain dead, (2) has an early myoclonus status or (3) if there are strong ethical reasons to withdraw intensive care. In the study protocol it is defined that: “the neurological evaluation will be based on a clinical neurological examination (including GCS motor score, pupillary and corneal reflexes), median nerve SSEP and EEG”. In all unconscious patients, a conventional EEG is performed at 12–36 hours after rewarming. SSEP is performed at 48–72 hours after rewarming at centers where this technique is available. In addition, information from neuroimaging (MRI and CT) may be used but cannot constitute the sole reason for withdrawal of intensive care. Biochemical markers for brain damage are not used for prognostication, instead serial blood samples are collected and stored in a central biobank for later analyses. The physician performing prognostication should make one of the following recommendations:

– Continue active intensive care

– Do not escalate intensive care

– Withdraw intensive care

Findings allowing for discontinuation of life support have been specified in the protocol (Table 1). In the case-record-form, findings from all examinations, recommendations and decisions are recorded.

**Table 1:** Findings allowing for discontinuation of life support in the TTM-trial

	**Time period**	**Findings**
1	Any time-point	Brain death.
2	<24 h from ROSC	Early myoclonus status^#^ and bilateral absence of N20 peak on SSEP after rewarming.
3	72 h after rewarming	GCS-M 1–2 and bilateral absence of N20 peak on SSEP performed 48–72 hours after rewarming, or later.
4	72 h after rewarming	A treatment refractory status epilepticus* and GCS-M 1–2.

# Generalized myoclonic seizures in face and extremities and continuous for a minimum of 30 min.

*Status epilepticus defined by EEG as sequences (>10 sec) of repetitive epileptiform discharges with an amplitude >50 μV and a medium frequency ≥1Hz, constituting >50% of a 30 minute period in a patient with or without clinical manifestations. Treatment refractory defined as unresponsive to treatment with propofol, midazolam or thiopental for at least 24 h in combination with at least one intravenous antiepileptic substance (including valproate and/or fos-Phenytoin) in adequate dose for at least 24 h. Free use of further antiepileptic substances and combinations at the discretion of the attending physician.

### Rationale for prognostication in the TTM-trial

Since we believe that the process of neurological prognostication may have an effect on the results of the study, we decided to blind the assessor for treatment temperature. However, the decision to continue or withdraw intensive care is multi-disciplinary and blinding is difficult to maintain throughout the prognostication process. However, as all recommendations and subsequent decisions are recorded, any systematic difference between the treatment arms will be detectable.

By strictly regulating the time for prognostication and criteria allowing for WLST, we have aimed to avoid false and premature predictions. The majority of patients with a favorable prognosis will wake up during the first three days after cardiac arrest [25] and we decided to postpone prognostication an extra 1.5 days to account for the effects of sedation during cooling and a possible delay of the recovery process by hypothermia. Recently published studies have shown that, although awakening itself may not be delayed by hypothermia [42], effects of sedation could explain why the clinical examination is less reliable [22]. Clinical examinations may still be unreliable at 4.5 days after cardiac arrest and data supporting this time-point is admittedly scarce [21]. Therefore, an extensor or absent motor response to pain at 4.5 days must be combined with 1) absent SSEPs or 2) a treatment refractory status epilepticus to allow for WLST in the TTM-trial. If any of these two conditions is not fulfilled, at least an extra day of observation is demanded.

We have defined minimal therapeutic efforts to assign a status epilepticus treatment as refractory, but we recognize that there might be a potential for recovery in some patients if more aggressive treatment and/or a longer observation time is allowed. Clearly, this is an area where more knowledge is urgently needed. The TTM-protocol defines when WLST is *allowed* but the treating physician makes the decisions and continued intensive care is always an option.

Many intensivists would consider it unethical to continue intensive care in a patient with early generalized and persistent myoclonus after cardiac arrest, but false predictions may occur both with [35] and without [43] hypothermia. We therefore combined an early myoclonus status with another strong predictor, absent cortical responses on the SSEP, and allowed for SSEP to be performed immediately after rewarming in patients with status myoclonus. Early prognostication is also allowed for the small fraction of patients who become brain dead and these patients should be diagnosed according to national legislation. We recommend, however, that the clinical diagnosis of brain death should be avoided during the first 24 hours after ROSC and be supported by radiological evidence of herniation and loss of intracerebral blood-flow when there is any doubt about the diagnosis [36]. Finally, strong ethical reasons for an early withdrawal of care may include generalized malignant disease or a clearly stated wish not to be resuscitated. Such reasons may become evident only after the patient has arrived in the ICU.

### Previous experience with prognostication at 72 hours after rewarming

As therapeutic hypothermia for cardiac arrest was introduced at the Skane University Hospital in Lund in 2003, a decision was made to postpone neurological prognostication for all patients who remain in coma until 72 hours after rewarming to account for a delayed recovery process. In a report of this strategy, 52% of hypothermia-treated patients awoke before the stipulated time for prognostication, 17% died early and 31% were still in coma 72 hours after rewarming (4.5 days after CA) [21]. Only 6/34 patients who were in coma at the time of prognostication awoke at some time-point and the rest remained in coma until death. In the majority of the deceased patients, clinical, neurophysiological, biochemical (neuron specific enolase (NSE)) and neuroradiologic findings supported a massive brain injury and this was confirmed by post mortem examinations. However, a sub-group of 8/34 patients with low-range NSE (≤20 μg/l) was identified, of whom 5/8 had normal MRI and 6/8 had normal SSEP. All these 8 patients had a generalized status epilepticus pattern on EEG and a lack of motor or extensor response to pain. Only one patient regained consciousness but was severely neurologically handicapped.

## Discussion

Despite international and national guidelines, the practice of neurological prognostication and WLST varies considerably and adheres poorly to recommendations [44,45]. Early prognostication in hypothermia treated cardiac arrest patients is associated with a high rate of false predictions of poor outcome [44]. It has been suggested that prognostication protocols used in previous clinical intervention trials on cardiac arrest patients, or rather the lack of such protocols, may have introduced bias and thus may have led to skewed results, since a delayed recovery process might have been missed [39].

Several recently published reviews deal with the issue of neuroprognostication after cardiac arrest in the era of hypothermia treatment and detail the reliability of individual prognostic instruments [46-49]. However, very little comparative data exist and it is still unclear whether the reported false predictions in hypothermia-treated patients were directly caused by cooling or other treatment measures such as altered principles for sedation. From the large amounts of comparative data in the TTM-trial, we will be able to learn how different prognostic instruments are affected by temperature. Until we learn more, it is the authors’ opinion that a reasonable clinical praxis is to delay prognostication further and avoid WLST-decisions based solely on clinical findings.

From a clinical trial perspective, the ideal strategy would be to refrain from prognostication and await the natural course of the post-anoxic encephalopathy but to our knowledge, this has never been done in a systematic way. Such an approach would have practical and ethical limitations. If a high level of care would be maintained and no treatment limitations implemented, many patients who remain in coma up to several days may risk surviving with a severe neurological handicap or in a chronic vegetative state.

Given the uncertainty about the optimal time for prognostication after cardiac arrest and the serious consequences if the window of opportunity for withdrawal of care is missed, the time-point of prognostication in a modern cardiac arrest-trial needs to be conservative enough to allow recovery of all patients with a favorable prognosis and strict enough to avoid unnecessary resource utilization and possible suffering.

## Conclusions

Principles for neurological prognostication and decisions on WLST are crucial for outcome after cardiac arrest and were not defined in the pivotal previous trials on therapeutic hypothermia. To reduce the risk of premature decisions to withdraw care, we recommend postponing neuroprognostication until at least 72 hours *after rewarming.* To further reduce the risk of bias, the assessor of prognosis should be blinded to treatment allocation. In our proposed model, a liberal use of adjunctive methods to support a decision on WLST is advocated. EEG has a central role to diagnose and adequately treat status epilepticus while SSEP may be used to allow for earlier withdrawal in patients with status myoclonus or confirm poor prognosis at 72 hours after rewarming. Neuroimaging is not a part of the model other than to give further support for a clinical brain dead diagnosis and to exclude other causes for coma than ischemic brain injury. The role of biomarkers for neuroprognostication after cardiac arrest is currently unclear and they are not a part of our proposed model.

## Competing interests

All authors are part of the steering group for the TTM-trial.

## Authors’ contributions

TC was the main writer but all authors contributed parts of the text and critically revised all versions of the manuscript. All authors read and approved the final manuscript.
